# Alteration of intestinal mucosal microbiota in mice with Chinese dampness-heat syndrom diarrhea by improper diet combined with high temperature and humidity environments

**DOI:** 10.3389/fcimb.2022.1096202

**Published:** 2023-01-04

**Authors:** Bo Qiao, Xiaoya Li, Maijiao Peng, Huaying Hui, Zhoujin Tan

**Affiliations:** ^1^ College of Chinese Medicine, Hunan University of Chinese Medicine, Changsha, China; ^2^ College of Pharmacy, Hunan University of Chinese Medicine, Changsha, China

**Keywords:** intestinal mucosal microbiota, improper diet, high humidity and temperature environment, Chinese dampness-heat syndrom diarrhea, metabolism

## Abstract

**Background:**

Environment, diet, and emotion may trigger diarrhea, but the mechanism is unclear. Dietary habits or environmental factors affect the composition of gut microbiota. This study aimed to investigate the effects of improper diet combined with high humidity and temperature (HTH) environment on the intestinal mucosal microbiota.

**Materials and methods:**

Kunming mice were randomly assigned to two equal groups of five mice, namely the control (ccm) group and the model (cmm) group. Diarrhea mice with dampness-heat (DSH) were established by improper diet combined with HTH environments. We used 16S rRNA gene amplicon sequencing to analyze the characteristics of intestinal mucosal microbiota and the interaction relationship of function.

**Results:**

Our study shows that the intestinal mucosal microbiota of mice changed significantly after an improper diet combined with the HTH environments. The abundance of Fusobacteria and *Haemophilus* increased dramatically in the cmm group compared to the ccm group (*P*<0.05). And the abundance of Firmicutes, *Lactobacillus*, and *Lonsdalea* was significantly decreased in the cmm group (*P*<0.05). According to the functional predictive analysis, we found that *Lactobacillus* showed a significant negative correlation with Protein export, Homologous recombination, Phenylalanine, tyrosine, tryptophan biosynthesis, Citrate cycle, and Lipoic acid metabolism.

**Conclusion:**

Diarrhea with DSH constructed under improper diet and HTH environment may be related to *Lactobacillus* and *Haemophilus*. And long-term consumption of improper diet and the HTH environment may affect metabolism.

## 1 Introduction

Traditional Chinese medicine (TCM) has suggested that the main reason for diarrhea with dampness-heat (DSH) syndrome is the dysfunctional bowel conductivity caused by the accumulation of hot and humid heat in the intestine (Yu et al., 2016; Li et al., 2021). This study simulated the traditional etiology of diarrhea with DSH from internal and external humidity combined with complex environmental and dietary factors. According to TCM, high sugar and fat (HSF) diet and high temperatures and high humidity (HTH) environment may lead to an accumulation of external and internal humidity in the intestine, whereas it impairs the spleen and stomach function in mice (Xiao et al., 2017; Wu et al., 2021). Combined gavage with ice water and alcohol further damages the spleen and stomach and causes diarrhea (Liu et al., 2021; Lu et al., 2021). In terms of TCM, there are adequate reasons for diarrhea with DSH caused by eating disorders and the HTH environment, but some evidence from modern medicine’s perspective is still needed.

The gut microbiota is the “second genome of the human body,” with about 10 trillion bacteria living in the gut (Matsumoto et al., 2012). The gut microbiota is directly or indirectly involved in immune regulation, energy supply, nutrient absorption, digestion absorption, fat metabolism, and disease prevention. In dysfunctional gut microbiota, large colonies of pathogenic bacteria play a role in the development of diarrhea (Ramirez et al., 2020). The gut microbiota in patients with ulcerative colitis (UC) centered on *Lactobacillus*, *Lactobacillaceae*, *Erysipelotrichaceae*, *Erysipelotrichales*, and *Akkermansia (* Ding et al., 2018). Restoring the microbiota through probiotics and fecal microbiota transplantation (FMT) has been dramatically welcomed among researchers in preventing and treating gastrointestinal diseases (Elangovan et al., 2019). Some researchers established diarrhea with DSH models by scientific and reasonable research methods and found that the abundance of Bacteroides and Proteobacteria increased significantly (Yao et al., 2017). Since different external factors may cause various changes in the body, the gut microbiota changes of diarrhea with the DSH leak model can also be multiple. Studies have found that changes in temperature and humidity and exposure to natural environments increase microbial diversity and alter community composition (Bär et al., 2020). Chen et al. found that the HTH environment directly leads to gut microbiota disorder and slight enteritis, whereas probiotics partially normalize the microbiota and reduce intestinal inflammation (Chen et al., 2019). Diet emerges as a critical determinant of gut microbiota community structure and function. Djésia et al. found that long-term consumption of a diet enriched with sucrose and fat predisposes mice to colitis (Arnone et al., 2021). Altogether, HSF diet-induced changes in host gut microbiota (Ojo et al., 2019; Horne et al., 2020; Gao et al., 2020; Guo et al., 2021).

**Figure 1:**
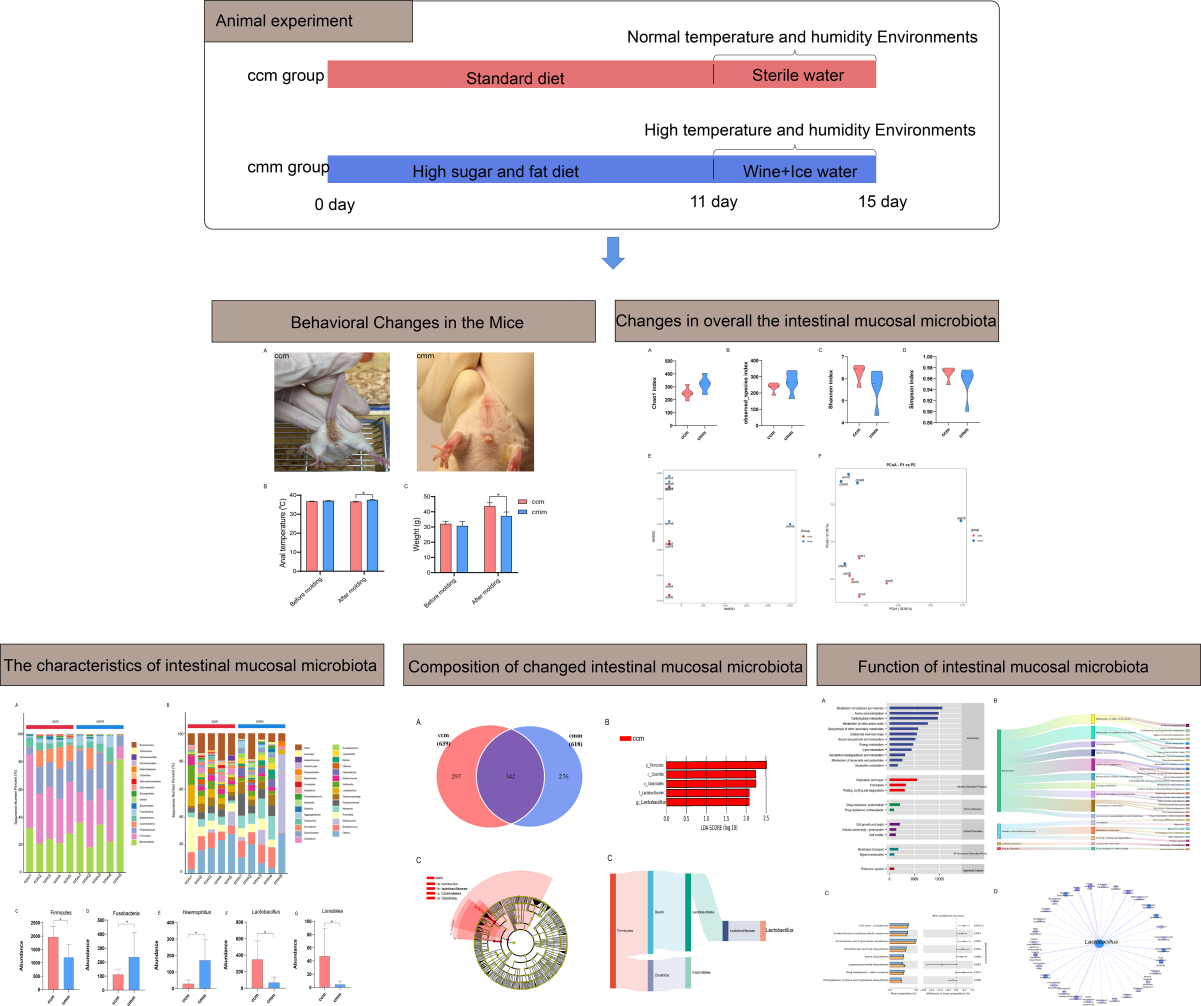
Experimental flow chart.

Meanwhile, these alterations in gut microbiota composition induced by diet were related to changes in secondary metabolite production, which promoted the development of host metabolic syndrome (Horne et al., 2020). The habit of drinking ice water leads to gastrointestinal diseases, most likely caused by an imbalance in the gut microbiota due to a drastic change in gastrointestinal temperature (Liang et al., 2020). There may be a correlation between the decreased surface temperature of the rat stomach and changes in intestinal microbiota after continuous and short-term cold stimulation (Guo et al., 2021). Several mechanisms have been shown to regulate the role of alcohol in the gastrointestinal tract (Patel et al., 2015). Alcohol could alter the gut microbiota, break down the gut barrier, increase intestinal permeability, and directly or indirectly boost immune activation (White et al., 2022). Our preliminary study reveals altered diversity and structure of gut content microbiota in diarrhea with DSH, especially *Lactobacillus gasseri* (Li et al., 2021). This study aims to explore the pathogenesis of diarrhea with DSH from the perspective of intestinal mucosal microbiota and provide a reference for the microecological mechanism of diarrhea with DSH. The specific process is shown in **Figure 1**.

## 2 Materials and methods

### 2.1 Experimental animals and grouping

Ten male (Wu et al., 2022) Kunming mice (20 ± 2g) were supplied by Slack Jingda Experimental Animal Co, Ltd. (license number: SCXK (Xiang) 2019-0009) and bred at the experimental animal center of Hunan University of Chinese Medicine (Changsha, China). The feeding conditions with specific pathogen-free and reared in line with standardized methods at a temperature of 23–25°C, a humidity of 50–70%, and a 12 h dark-light cycle. The experiment complied with the standards of the Animal Ethics and Welfare Committee of Hunan University of Chinese Medicine.

After a three-day acclimatization period, ten male mice were randomly divided into the control (ccm) group and the model (cmm) group, with five mice in each group. Intervention on mice referring to previous methods (Li et al., 2021), the cmm group was fed with a high-sugar and high-fat (ordinary feed 80% mixed with 12% lard and 8% honey, Jiangsu Synergetic Pharmaceutical Biological Engineering Co., LTD.) diet for 11 days. From the 11th day of modeling, the mice in the cmm group were placed in an artificial climate chamber at 32 ± 0.5 °C and 95% relative humidity for eight h•d^-1^ for seven days. And the mice were gavaged with liquor (brand: Red Star Erguotou, production license number: SC11511160310087) diluent 10 mL/kg (V liquor: V sterile water=1:1) and at 0°C ice water 10 mL/kg, twice a day, for four days. The ccm group was routinely fed and gavaged with an equal amount of distilled water.

### 2.2 General behavioral observations

We observed the behavioral status of mice in the ccm and cmm groups on day 15 of the modeling period. And we recorded the mice of fecal characteristics, body weight, and anal temperature before and after molding.

### 2.3 Intestinal mucosal collection

After removing the contents from the chyle, the intestinal wall was flushed with saline. Scrape the intestinal mucus with a coverslip and add twice the amount of saline to the solution. Then it was centrifuged at 3000 r/min for 10 minutes, and the supernatant was taken for the subsequent extraction of genes (Long et al., 2020; Qiao et al., 2022).

### 2.4 16S rRNA gene high--throughput sequencing

Each group selected five intestinal mucosal samples for 16S rRNA sequencing (Li et al., 2022). Nuclein was extracted using a NanoDrop ND-2000 spectrophotometer (Thermo Fisher Scientific, Waltham, MA, USA). 0.8% agarose gel electrophoresis was used to judge the molecular size. And the DNA was quantified by ultraviolet spectrophotometer. The forward primer 27F (5’-AGRGTTYGATYMTGGCTCAG-3’) and reverse primer 1492R (5’-RGYTACCTTGTTACGACTT-3’) were used for PCR amplification of bacterial 16S rRNA gene. The amplification results were subjected to 2% agarose gel electrophoresis, and the target fragments were cut out and then recovered with the Agencourt AMPure Beads (Beckman Coulter, Indianapolis, IN). The PCR products are quantified through the PicoGreen dsDNA assay kit (Invitrogen, Carlsbad, CA, USA) and mixed according to each sample’s required data. The library was constructed using SMRTbell Template Prep Kit 1.0-SPv3. For qualified libraries, paired-end sequencing used the PacBio platform with DNA/Polymerase Binding Kit 3.0 (PacBio) at Frasergen Genomics Information (Wuhan, China).

### 2.5 Bioinformatics and statistical analysis

Effective sequences were clustered into OTUs with 97% similarity, and representative OTUs were identified by classification. Calculate the Chao1 index, Observed species index, Shannon index, and Simpson index to compare the richness and average among different samples. Beta diversity analysis was performed to investigate the structural variation of microbial communities across samples using UniFrac distance metric and visualized *via* principal coordinate analysis (PCoA) and nonmetric multidimensional scaling (NMDS) (Bray and Curtis, 1957). Detect groups with significant differences and identify potential biomarkers by linear discriminant analysis (LEfSe) (Segata et al., 2011). Microbial functions were predicted by PICRUSt2 upon KEGG (https://www.kegg.jp/) statistical database analysis. Based on the correlation coefficient and significance, we construct the modular network.

Data expressed as mean ± standard deviation. When the data conformed to the normal distribution and the homogeneity of variances was satisfied, the differences among multiple groups were compared and analyzed by an independent sample T-test. *P*<0.05 was considered significant.

## 3 Results

### 3.1 Behavioral changes in the mice

**Figure 2:**
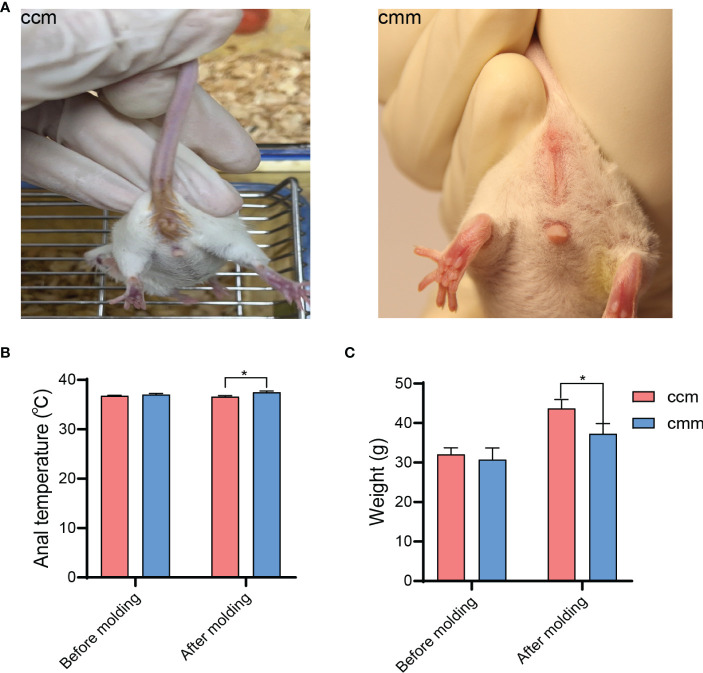
Behavioral changes in the mice. **(A)** Perianal of the mice. **(B)** The anal temperature of the mice. **(C)** Body weight **(g)** of the mice. ccm group, control group; cmm group, model group (*n* = 5). The values were expressed as mean ± standard deviation. * p < 0.05.

As visible from **Figure 2A**, the mice in the ccm group had a normal mental state and autonomous activity, were responsive, with smooth fur, and clean perianal area. The mice in the cmm group were unresponsive, and the fur was pale, the feces was not shaped, and the fecal attachment was around the anus. We found that the difference in body weight between the mice in the ccm and cmm groups was not significant before modeling. On the 15th day of modeling, the body weight of the mice in the cmm group was significantly lower compared with that of the ccm group (*P*< 0.05) (**Figure 2B**). And after modeling, the anal temperature of the mice in the ccm group was significantly lower than that in the cmm group(*P*< 0.05)(**Figure 2C**). These results indicated that the improper diet combined with HTH environment-induced diarrhea had suppressive effects on the body weight of mice.

### 3.2 Changes in overall the intestinal mucosal microbiota of improper diet combined with HTH environment

**Figure 3:**
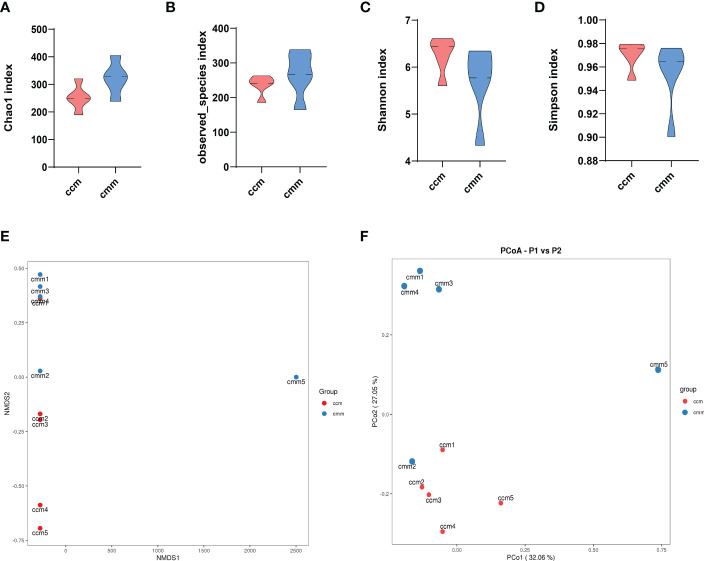
The diversity index for all three groups of animals. **(A–D)** Chao1 index, Observed_species index, Shannon index, Simpson index. **(E)** NMDS analysis. **(F)** PCoA analysis. ccm group, control group; cmm group, model group (*n* = 5).

As shown in **Figures 3A, B**, Chao1 and Observed_species indexes were slightly higher in the cmm group than in the ccm group (*P >*0.05), while the Simpson and Simpson indexes showed decreasing trends in the cmm group (*P >*0.05) (**Figures 3C, D**). This study evaluated beta diversity by NMDS and PCoA analysis (**Figures 3E, F**). NMDS reflects the information of the distance matrix between samples. The contribution rate of the abscissa PCo1 was 32.05%, and the contribution rate of the ordinate PCo2 was 27.05%. The ccm and cmm samples were efficiently separated and presented the phenomenon of grouping and aggregation. The results illustrate that the composition and abundance of intestinal mucosal microbiota changed after an improper diet combined with the HTH environment.

### 3.3 Composition of changed intestinal mucosal microbiota of improper diet combined with HTH environment

**Figure 4:**
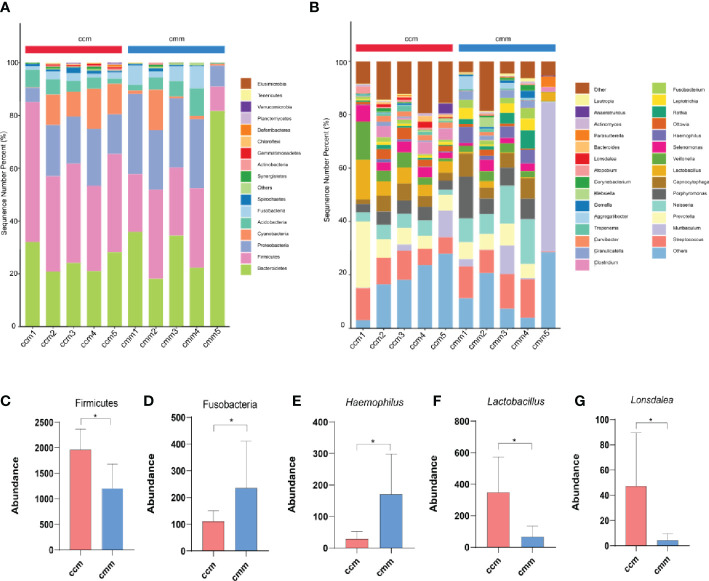
Composition analysis of intestinal mucosal microbiota. **(A)** Intestinal mucosal microbiota composition in the phylum. **(B)** Intestinal mucosal microbiota composition at the genus. **(C–G)** Phylum and genus levels of dominant bacteria. ccm group, control group; cmm group, model group (*n* = 5). The values were expressed as mean ± standard deviation. * p < 0.05.

The results showed that the dominant phyla of the mouse were Bacteroidetes, Firmicutes, Proteobacteria, Cyanobacteria, Actinobacteria, Fusobacteria (**Figure 4A**). *Streptococcus*, *Muribaculum*, *Neisseria*, and *Lactobacillus* were the dominant bacteria in the two groups (**Figure 4B**). The ccm group has a much higher abundance of Firmicutes than the cmm group, as shown in **Figure 4C** (*P*<0.05). And the abundance of Fusobacteria in the cmm group was markedly higher than in the ccm group (*P* < 0.05)(**Figure 4D**). The abundance of *Haemophilus i*n the cmm group was higher than in the ccm group (*P* < 0.05)(**Figure 4E**). The abundance of *Lactobacillus* and *Lonsdalea* in the ccm group was markedly higher than in the cmm group (*P* < 0.05)(**Figures 4F, G**). These results suggest that an improper diet combined with HTH Environment alters the relative abundance of intestinal mucosal microbiota.

### 3.4 The characteristics of intestinal mucosal microbiota of mice with improper diet combined with HTH environment

**Figure 5:**
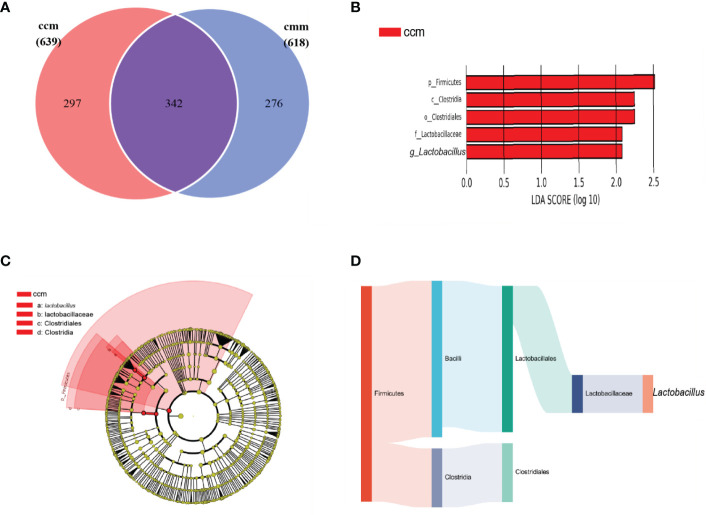
The characteristics of intestinal mucosal microbiota of mice. **(A)** Venn diagram. **(B)** LDA scores Chart. **(C)** Cladogram diagram. **(D)** Sankey diagram (From left to right are phyla, class, order, family, genus). ccm group, control group; cmm group, model group (*n* = 5).

The ccm group had 639 OTUs with 297 unique OTUs; the cmm group had 618 OTUs with 276 unique OTUs (**Figure 5A**). LEfSe analysis identified five differentially altered bacterial signature taxa (LDA scores > 2), indicating significant structural differences between the two groups (**Figure 5B, C**). The abundance of Firmicutes, Bacilli, Lactobacillates, Lactobacillceae, *Lactobacillus*, Clostridia, and Clostridialesp was markedly enhanced in the ccm group, which played an essential role in diarrhea with DSH. Analysis of the superordinate taxonomic level of *Lactobacillus* from the ccm group revealed (**Figure 5D**) that from the phylum level to the family level as Firmicutes, Bacilli, Lactobacillates, and Lactobacillceae. The above shows that *Lactobacillus* played an essential role in diarrhea with DSH.

### 3.5 Effects of improper diet combined with HTH environment on the function of intestinal mucosal microbiota in mice

**Figure 6:**
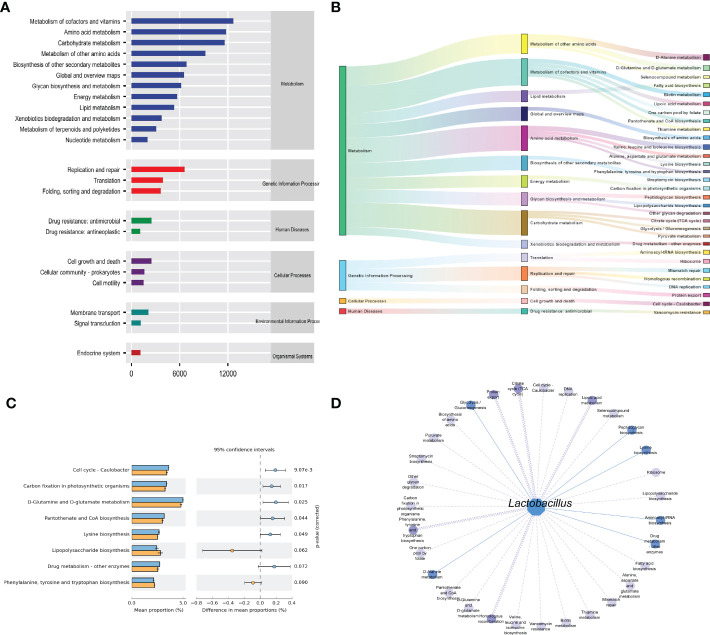
Functional analysis of intestinal mucosal microbiota. **(A)** Predictive abundance diagram of function. **(B)** Sankey diagram. **(C)** Predictive abundance diagram of function. **(D)** Interaction network of “*Lactobacillus*-function.” Solid lines indicate a positive correlation, and dotted lines are negative correlations. The line width stated the strength of the correlation.

The functions of intestinal mucosal microbiota are generally divided into six categories. The second level includes 23 sub-function categories, with the metabolic function accounting for a greater abundance and the metabolic function containing 46 categories (**Figure 6A**). The three-level included 331 sub-functional categories, of which the median value was >1130.681, including 31categorie (**Figure 6B**). As shown in **Figure 6C**, the cmm group significantly influences D-Glutamine and D-glutamate metabolism, Cell cycle-Caulobacter, and Carbon fixation in photosynthetic organisms. In the gut micro-ecosystem, gut mucosal microbiota and metabolic functions play a crucial role in maintaining the stability of the microenvironment, but their functional relevance is unclear. As a result, *Lactobacillus* showed functional correlation, including six existing positive correlations and 25 pairs of existing negative correlations (**Figure 6D**). The strongest negative correlation was with Lipoic acid metabolism, Protein export, Homologous recombination, Citrate cycle (TCA cycle), Phenylalanine, tyrosine, and tryptophan biosynthesis. In conclusion, the above-mentioned metabolic functions and pathways may be the main pathways affecting mice’s changes in intestinal mucosal microbiota.

## 4 Discussion

In this study, we established diarrhea with DSH in mice by improper diet combined with high temperature and humid environments. HSF diet may damage the function of the spleen and stomach, cause a disorder of functional activities of vital energy, and induce internal humidity and internal heat (Chen et al., 2017). HTH environment may lead to spleen and stomach dysfunction by increasing “external dampness to induce internal dampness.” Liquor is a good combination of hot and humid and has the function of clearing heat and humidity. Ice water can cause endogenous moist heat, cold abdominal pain, diarrhea, and other symptoms. In this experiment, the modeling methods and the results of symptoms in the model mice were consistent with the diagnostic criteria of DSH, indicating that the model was successfully replicated (Hui et al., 2021). The main clinical sign of diarrhea with DSH is closely related to gut microbiota dysbiosis (Gong et al., 2015; Yan et al., 2022). Studies have shown that digestive tract diseases occur primarily due to structural changes in bacteria of typical origin (Sorbara and Pamer, 2022).

Gut microbiota plays a vital role in human health. The number of microorganisms inhabiting the gastrointestinal tract has been estimated to exceed 1013. The dominant genera in the human gut are Firmicutes (more than 180 species of *Lactobacillus*), Actinobacteria (among others, the *Bifidobacteria*), Bacteroidetes (the most important is *B. fragilis*) and Proteobacteria (*E. coli*, *Salmonella*, *Haemophilus*, etc.). Still, the microbiota composition varies concerning host genetics, stress, diet, antibiotics, and early childhood experiences (Sabo and Dumitrascu, 2021). Researchers have tried to uncover the relationship between gut microbiota and disease, predict disease likelihood based on the type and number of gut microbiota, and determine disease severity. Our research showed that the OTU number of intestinal mucosal microbiota in the cmm group slightly decreased. The Chao1 index and the observed species index in the alpha diversity index increased, while the Shannon and Simpson index decreased slightly. The results indicated that diarrhea with DSH had no apparent effect on the intestinal mucosal microbiota diversity. According to the PCA and NMDS, the sample distribution of the cmm and ccm groups differs. Therefore, we conclude that changes in diet and environment alter the structure of intestinal mucosal microbiota.

The composition and abundance of the intestinal mucosal microbiota were the focus of this study. We found that the abundance of Bacteroidetes, Firmicutes, Proteobacteria, Cyanobacteria, Actinobacteria, and Fusobacteria occupied the dominant position in the phylum. And the abundance of Fusobacteria increased, and the abundance of Firmicutes decreased after an improper diet combined with the HTH environment. At the genus, the abundance of *Lactobacillus* and *Lonsdalea* decreased in the cmm group. A further study found that the abundance of *Lactobacillus* was significantly reduced in the cmm group, indicating that diarrhea with DSH inhibited the growth of *Lactobacillus*, the significant decrease in the abundance of *Lactobacillus* may be the main reason for the occurrence of diarrhea with DSH. *Lactobacillus* plays functional roles in the human body, for example, converting sugars to lactic acid (Yang et al., 2018). *Lactobacillus* is widely found in the human oral cavity, gastrointestinal tract, and genitourinary tract, which are often used as probiotics and can benefit host health when administered in adequate amounts (Thorlacius et al., 2003; Macfarlane and Dillon, 2007; Gagliardi et al., 2018). *Lactobacillus* adherent to the surface of intestinal mucosal cells is the primary condition for colonization (O'Callaghan and O'Toole, 2013). *Lactobacillus reuteri DSM 17938* (*L. reuteri*) is a probiotic that can colonize different human body sites, including primarily the gastrointestinal tract, the urinary tract, the skin, and breast milk. Numerous clinical studies suggested that *L. reuteri* may help modulate gut microbiota, eliminate infections, and attenuate the gastrointestinal symptoms of enteric colitis, antibiotic-associated diarrhea, irritable bowel syndrome, inflammatory bowel disease, and chronic constipation (Saviano et al., 2021). *Haemophilus* is a Gram-negative bacterium that causes primary septic and secondary infections (Wong and Akerley, 2012). *Haemophilus* mainly resides in the throat, and oral mucosa of humans and animals, and a few are found in the reproductive tract. It can cause primary suppurative infection and severe secondary infection. Liu et al. found that *Haemophilus* increased in the feces of patients with IBS-D (Liu et al., 2020). We observed that mice developed diarrhea symptoms that could be associated with *Lactobacillus* and *Haemophilus* after the intervention.

**Figure 7:**
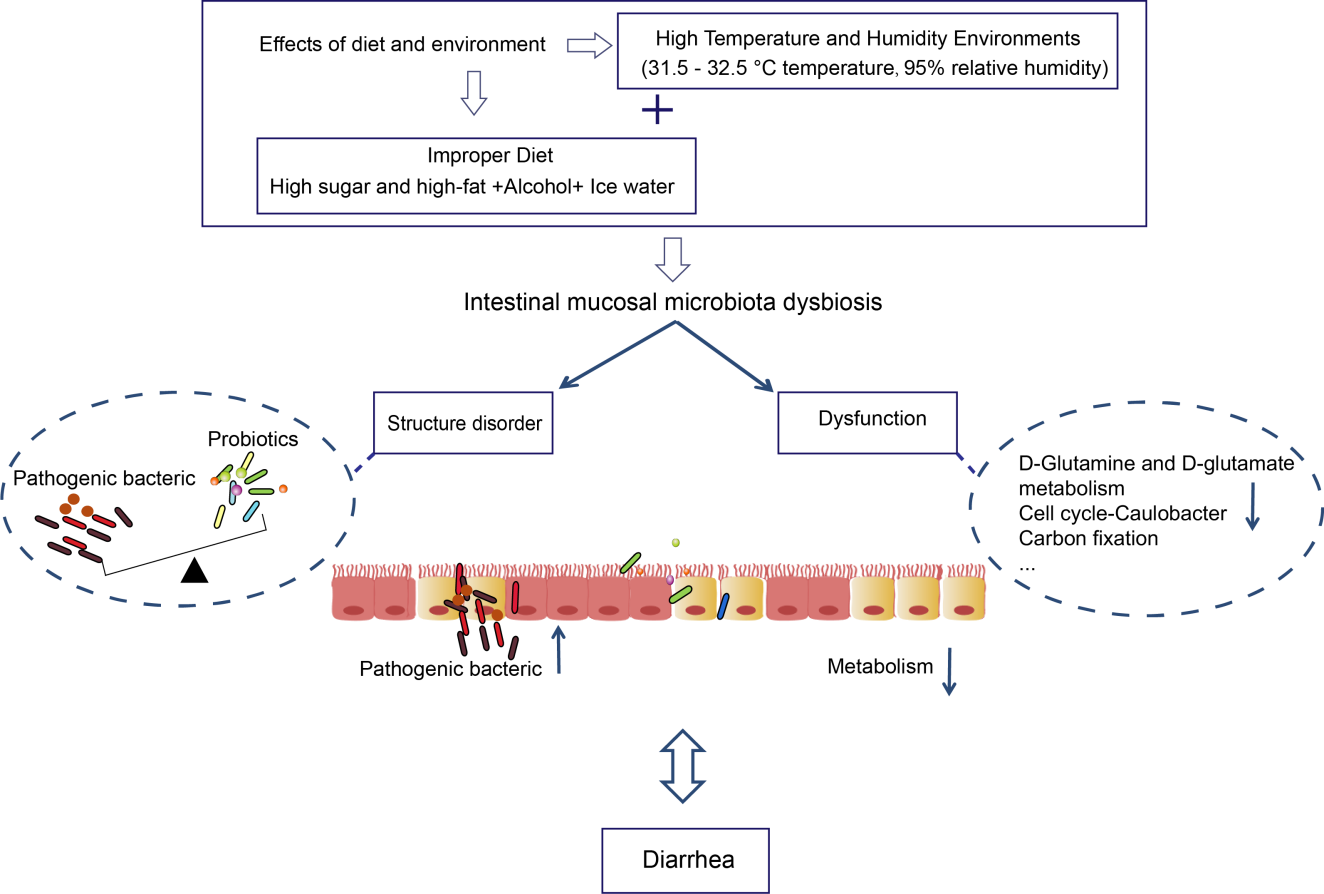
The role of intestinal mucosal microbiota in diarrhea caused by improper diet combined with HTH environment.

Moreover, we found that the metabolic function of intestinal mucosal microbiota accounted for a more significant proportion of the functional prediction. And *Lactobacillus* has the strongest negative correlation with Lipoic acid metabolism, Protein export, Homologous recombination, Citrate cycle (TCA cycle), Phenylalanine, tyrosine, and tryptophan biosynthesis. Lipoic acid is a significant cofactor in mitochondrial metabolism, and mitochondria can synthesize fatty acids in a malonyl-CoA/acyl carrier protein (ACP)-dependent manner (Solmonson and DeBerardinis, 2018). El-Gowelli et al. found that α-lipoic acid and cyclosporine protect significantly against acetic acid-induced ulcerative colitis (El-Gowelli et al., 2015). Dadhania et al. reported that α-lipoic acid pretreatment ameliorates methotrexate-induced intestinal toxicity (Dadhania et al., 2010). Furthermore, lipoic acid is crucial in protecting the gastrointestinal. The citric acid cycle is the final common oxidation pathway of carbohydrates, fats, and amino acids. Liubets’ka et al. found that peculiarities of redox processes of glycolysis and citrate cycle in newborn calves under oxygen shortage have been caused by diarrhea under acute digestion disturbances. Hydrogen peroxide is the most important metabolic pathway for the energy supply (Packer et al., 1995). The tricarboxylic acid (TCA) cycle is the most critical central pathway connecting almost all metabolic pathways and essential metabolites for biosynthetic reactions (Arnold et al., 2022). Citrate is a necessary component of the TCA cycle, a substrate for fatty acid biosynthesis and sterols, and a key regulator of intermediate energy metabolism (Parkinson et al., 2021). We found that the metabolic processes of sugar, fat, protein, and nucleic acid changed after an improper diet combined with HTH environments. All in all, the intestinal mucosal microbiota disorder may be one of the mechanisms of action of improper diet combined with HTH environment-induced diarrhea (**Figure 7**).

## 5 Conclusion

In summary, improper diet combined with HTH environments may synergize with Fusobacteria and *Haemophilus*. And the relative abundance of Firmicutes, *Lactobacillus*, and *Lonsdalea* was significantly decreased. *Lactobacillus* showed a significant negative correlation with Protein export, Homologous recombination, Phenylalanine, tyrosine, tryptophan biosynthesis, TCA cycle, and Lipoic acid metabolism. According to bacterial function prediction and correlation analysis, long-term consumption of improper diet and the HTH environment may affect metabolism. At the same time, our research is somewhat limited. And we used a 16S rRNA amplitude rather than metagenomic sequencing, which limited our ability to find specific diet-related bacteria at the species level. And we should consider the influence of single factors on the intestinal mucosal microbiota.

## Data availability statement

The datasets presented in this study can be found in online repositories. The names of the repository/repositories and accession number(s) can be found in the article/Supplementary Material.

## Ethics statement

The animal study was reviewed and approved by the Animal Ethics and Welfare Committee of Hunan University of Chinese Medicine. Written informed consent was obtained from the owners for the participation of their animals in this study.

## Author contributions

ZT was responsible for studying the design and collecting funds. BQ and XL collected the data. BQ analyzed the data and drafted the manuscript. MP and HH guided the performance of the animal experiment. All authors reviewed and approved the final manuscript. All authors contributed to the article and approved the submitted version.
